# The Importance of mTOR Trafficking for Human Skeletal Muscle Translational Control

**DOI:** 10.1249/JES.0000000000000173

**Published:** 2018-12-14

**Authors:** Nathan Hodson, Andrew Philp

**Affiliations:** 1 School of Sport, Exercise and Rehabilitation Sciences, University of Birmingham, Birmingham, UK; and; 2 Garvan Institute of Medical Research, Darlinghurst, Sydney, New South Wales, Australia

**Keywords:** mTORC1, lysosome, resistance exercise, amino acids, skeletal muscle

## Abstract

The mechanistic target of rapamycin (mTOR) is a central regulator of muscle protein synthesis, and its activation has long been attributed to its translocation to the lysosome. Here, we present a novel model of mTOR activation in skeletal muscle where the translocation of mTOR and the lysosome toward the cell membrane is a key process in mTOR activation.

## Abstract 2

This review will critique cell, rodent, and human models of mTOR regulation to discuss why mTOR trafficking may represent a novel and physiologically relevant model of regulation in skeletal muscle.

**Key Points**

- Cell and murine models suggest that movement of mechanistic target of rapamycin (mTOR) to the lysosome is a fundamental processes enhancing mRNA translational capacity.
- Recent work from our laboratory suggests that translocation of mTORC1/lysosomal complexes toward the cell membrane is a key event in mTORC1 activation after resistance exercise and amino acid ingestion in human skeletal muscle.
- mTORC1/lysosomal complex translocation facilitates mTORC1 interaction with upstream activators (Rheb), translation initiation factors (eIF3F), and the microvasculature during a period of increased protein synthesis.

## INTRODUCTION

This paper presents the novel hypothesis that the translocation of mTORC1/lysosomal complexes to the cell membrane is a critical factor driving the initial phase of protein translation in human skeletal muscle after resistance exercise or amino acid ingestion.

### The Importance of Protein Balance for Skeletal Muscle Regulation

Skeletal muscle is a highly plastic tissue, displaying an ability to both grow and decrease in size and structure regularly throughout lifespan. The control of skeletal muscle homeostasis is provided through the balance of two dynamic processes, skeletal muscle protein synthesis (MPS) and muscle protein breakdown (MPB), with each varying significantly on a day-to-day basis (1,2). After ingestion of amino acids/protein, the greater amounts of substrates available for MPS, and the activation of signalling pathways, cause MPS to rise (2), whereas the insulinogenic effects of amino acids and other constituents of meals (carbohydrates) elicit a slight suppression of MPB (3). This culminates in a time period where MPS exceeds MPS and net protein balance (NPB) is positive. In these periods, muscle proteins will be accreted. In postabsorptive states, when substrates for MPS are not readily available, MPS will lower and MPB (ubiquitin-ligase and autophagic systems) will increase to provide any needed amino acids and remove damaged/dysfunctional proteins (4). During these periods, MPB will surpass MPS, causing a negative NPB and muscle protein loss. In individuals who have a reasonably active lifestyle and a healthy, balanced diet, these periods of net muscle gain and loss, throughout a day, will be equal therefore causing muscle mass maintenance.

An exercise stimulus, both aerobic and resistive in nature, also stimulates MPS (5,6), although the extent of this is much greater after resistance exercise. In addition, such stimuli elicit increases in MPS such that exercise conducted in the fasted/postabsorptive state, with no postexercise nutrients ingested, only serves to allow NPB to become less negative (6). However, exercise does serve to sensitize the muscle to nutrients, and an increase in the amount of amino acids available postexercise, that is, via a protein beverage, will cause a rise in MPS that is greater than that stimulated by either exercise or nutrients alone (7). Again, a slight suppression of MPB is elicited via the effects of insulin (3), causing NPB to become positive and the muscle enters a state of protein accretion. If this process is repeated regularly such that daily NPB is frequently positive, then, over time, skeletal muscle hypertrophy likely will occur (8).

### mTOR Is a Central Regulator of MPS in Skeletal Muscle

At the center of the regulation of skeletal muscle, MPS is the mechanistic target of rapamycin (mTOR). This evolutionary conserved serine/threonine kinase resides in two complexes, each exhibiting unique roles (9). mTOR complex 1 (hereafter referred to as mTORC1) is composed of mTOR, regulatory-associated protein of mTOR (RAPTOR), DEP domain-containing mTOR-interacting protein (DEPTOR), proline-rich AKT substrate 40 kDa, and G-protein beta subunit-like (GβL) (9) and is involved in cellular growth (10). In contrast, mTOR complex 2 contains the rapamycin-insensitive companion of mTOR (RICTOR), mammalian stress-activated protein kinase interacting protein 1, PROTOR-1/2, DEPTOR, and GβL and coordinates actin cytoskeleton dynamics and glucose uptake (9). Due to its roles in growth regulation, mTORC1 is the mTOR complex that is believed to be primarily associated with the control of skeletal muscle NPB (11). Intensive research has therefore focused on understanding mTORC1’s downstream activity and how this complex can stimulate skeletal muscular hypertrophy in response to anabolic stimuli (resistance exercise and protein feeding).

*In vitro* studies comprehensively have characterized mTORC1 substrates and their roles in cellular growth. The most well-known mTORC1 targets are p70 S6 kinase 1 (S6K1) and eukaryotic translation initiation factor 4E-binding protein 1 (4EBP1), both of which are phosphorylated in an mTORC1-dependent manner in many cell types under nutrient-rich conditions (12,13). The phosphorylation of S6K1 activates its kinase capabilities, allowing it to phosphorylate ribosomal protein S6, a component of the 40S ribosomal subunit, and activate protein translation (14). S6K1 also is known to have other targets for phosphorylation including S6K1 Aly/REF-Like Target and eukaryotic translation initiation factor 4B (15), both of which also enhance protein translation. mTORC1-dependent phosphorylation of 4EBP1 exhibits inhibitory effects, removing this protein from its association with eukaryotic translation initiation factor 4E permitting translation initiation to occur (15). These molecular events are corroborated in studies of rodent and human skeletal muscle displaying mTORC1s regulatory role in MPS (10,16). In fact, the vital importance of mTORC1 activity to MPS has been shown elegantly in humans through the use of the mTORC1-specific inhibitor rapamycin. Ingestion of this compound before commencing resistance exercise or the ingestion of amino acids completely can ablate the effects of these anabolic stimuli on MPS, suggesting mTORC1 to be critical in these processes (17,18).

In addition to stimulatory effects on protein translation/accretion, mTORC1 also exerts inhibitory effects on the second component of skeletal muscle NPB, protein catabolism. When active, mTORC1 phosphorylates proteins involved in the autophagic (UV radiation resistance-associated gene protein, unc-51 like autophagy activating kinase (ULK1), and transcription factor EB (TFEB)) and ubiquitin-proteasome (extracellular signal-regulated kinase 5) systems (15), inhibiting their effects on autophagosome formation and proteasome assembly, respectively. Such events act to reduce protein breakdown in cells, allowing NPB to shift toward net protein accretion. These processes are less well characterized *in vivo* with varying findings reported; however, constitutively active mTORC1 seems to cause myopathy because of autophagy inhibition through the phosphorylation of ULK1 (19). Taken together, these data demonstrate the bidirectional control of NPB, by mTORC1, and show how this kinase complex is essential in the regulation of this dynamic process in skeletal muscle.

### mTOR Association With the Lysosome Is Essential for mTORC1 Activation

Lysosomes are spherical organelles that contain a variety of hydrolytic enzymes that digest redundant components of the cell, that is, damaged proteins, with these digested materials then available to be used in the production of new cellular components (20). Seminal work from the Sabatini lab (21) using immunofluorescence microscopy displayed that mTORC1, when activated under nutrient-rich conditions, localized with late endosomes/lysosomes (Ras-related protein Rab7 positive staining). This work suggested that mTORC1 requires lysosomal association to become maximally activated. Further reports have since reinforced this notion. For example, forced targeting of mTORC1 to the lysosomal surface in HEK293 cells continuously activates the kinase, renders this pathway amino acid insensitive, and results in cellular hypertrophy (22). Such protocols also have been used *in vivo*, with the mutant RAPTOR-Rheb (Ras homolog enriched in brain) construct, which anchors mTORC1 to the lysosome, transfected into rodents (23). Here, similar observations were reported, as this process elicited a fivefold elevation in S6K1^T389^ phosphorylation, a commonly used readout of mTORC1 activation. The disruption of normal lysosomal function also inhibits mTORC1 activation in response to anabolic stimuli *in vitro* (24), as well as altering gene expression profiles in response to essential amino acid ingestion *in vivo* (25). As such, there is strong evidence to suggest that mTORC1-lysosomal association is essential to this complex’s activation both *in vitro* and *in vivo*.

In addition to being a docking site for mTORC1, the lysosome also supports two known activators of mTORC1, Rheb and phosphatidic acid (PA), which have been shown to be enriched in lysosomal membranes (26,27). These mTOR activators can bind directly to domains on the mTOR kinase and increase mTORC1 phosphorylation of downstream targets (28,29). In addition to the presence of these activators, the lysosome also contains an abundance of amino acids produced through the digestion of unwanted cellular proteins. These amino acids both activate mTORC1 (13) and are used as substrates in protein translation. Thus, it seems the lysosome is an ideal location for mTORC1 to become optimally activated.

**Figure 1:**
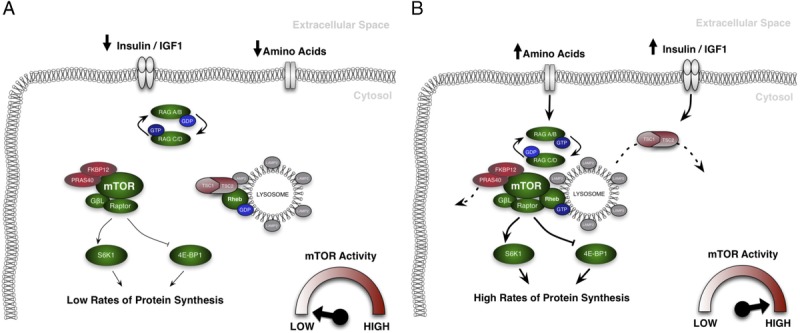
Depiction of the current, most widely accepted model of mechanistic target of rapamycin complex 1 (mTORC1) activation. When nutrient levels within a cell are low, mTORC1 resides away from the lysosome. As it is not in contact with its direct activators (Ras homolog enriched in brain (Rheb) and phosphatidic acid (PA)), and away from the abundant supply of amino acids that the lysosomes provide, its activity is low. This results in low phosphorylation levels of key mTORC1 substrates S6K1 and 4EBP1, and consequently protein synthesis is diminished (A). When nutrients are introduced to the cell, the guanosine-triphosphate (GTP)-loading status of the Rag heterodimer is altered, resulting in its recruitment to the lysosome. This in turn recruits mTORC1 to the lysosome, while simultaneously TSC2 is phosphorylated and removed from its association with Rheb. As mTORC1 can now bind to its direct activators, its activity increases, elevating phosphorylation of downstream targets and causing an increase in protein synthesis (B).

The mechanism by which mTORC1 is translocated to the lysosome appears to involve an interplay with Rag family of small GTPase proteins. Co-immunoprecipitation experiments in HEK293 cells have shown mTORC1 to associate with Rag GTPase proteins in cells upon amino acid introduction (21,22). These proteins reside at the lysosome in a heterodimer of RagA/B bound to RagC/D (21) and are only able traffic mTORC1 to the lysosome when RagA/B is guanosine-triphosphate (GTP)-loaded and Rag C/D guanosine-diphosphate (GDP)-loaded through association with the RAPTOR component of mTORC1 (22). Expression of dominant-negative mutants of these proteins prevents mTORC1-lysosome association and abolishes mTORC1 activity even if amino acid availability is high (21). This status is governed by the activity of other proteins situated at the lysosome, such as the ragulator complex, which acts as both a scaffold for Rag proteins to the lysosome (22) and a guanine nucleotide exchange factor to Rag A/B, initiating the change from GDP- to GTP-loading upon activation by amino acids (30). The GTP-loading status of RagC/D is controlled by folliculin, a tumor suppressor protein that has GTPase-activating protein (GAP) activity toward RagC/D (31) and increases its GDP-loaded status upon amino-acid activation. The intricate network of mTORC1 trafficking to the lysosome in HEK293 cells has been proposed recently to be coordinated by the activity of the v-ATPase, a vacuolar-associated ATP hydrolase that senses intralysosomal amino acids and signals to the ragulator-Rag complex to ultimately associate mTORC1 to the lysosomal surface (32). *In vivo* work has supported this mechanism in rodent skeletal muscle, where eccentric contractions enhanced mTOR-lysosome colocalization in concurrence with enhanced S6K1^T389^ phosphorylation (27). This model of mTORC1 activation is depicted in Figure 1, displaying the dissociation of mTORC1 from the lysosome when nutrient levels are low (Fig. 1A) and subsequently the translocation of mTORC1 to the lysosome upon nutrient introduction/mechanical stimulation (Fig. 1B). Overall, these data suggest that mTOR-lysosomal association is essential for the optimal activation of mTORC1 to both nutrients and mechanical loading.

### Is the Cellular Localization of mTOR-Lysosomal Complexes Biologically Relevant?

As discussed, several cell and rodent-based investigations have suggested that the recruitment of mTORC1 to the lysosome is a critical factor in mTORC1 activation in response to elevated amino acid availability (21,22,30,31). However, more recently, a role for the translocation of mTORC1-lysosome complexes has been proposed as an additional layer of mTORC1 activation (33). Korolchuk *et al.* (33) first proposed this hypothesis after the observation that physiologically relevant amino acid deprivation, milder in nature to that previously used (21,32), did not result in mTOR disassociation from the lysosome yet reduced mTORC1 activity in HeLa cells. This notable observation suggests that under physiological nutrient-deprived conditions, that is, the postabsorptive period in human skeletal muscle when autophagy and protein breakdown increase to maintain intracellular amino acid concentration (34,35), mTORC1 activity may not be governed by lysosomal association. After this, the authors reported an association between the number of cells with predominantly peripheral lysosomes, measured through immunofluorescence microscopy, and the extent of mTORC1 activation after amino acid stimulation. This notion was then tested more directly through the use of nocodazole, a drug that depolymerizes microtubules and prevents lysosomal movement. When this drug was administered to cells, the response of mTORC1 activity to nutrient reintroduction after deprivation was abolished (33). Further investigation through the use of siRNA targeting a protein implicated in lysosomal movement, ADP-ribosylation factor-like protein 8B, confirmed these observations. The knock down of this protein fixed lysosomes close to the nucleus of HeLa cells and prevented S6K1^T389^ phosphorylation irrespective of intracellular amino acid levels (33). Moreover, when this protein was overexpressed in HeLa cells, the amount of peripheral lysosomes were raised by 350%, but mTORC1 activity was elevated only when amino acid concentrations were sufficient (33). These data have been replicated in the osteosarcoma cell line U2OS (36) expressing an increased activity of the transcription factor E2F1. Upon activation of E2F1, lysosomal-associated membrane protein 2 (LAMP2)–positive structures (lysosomes) were seen to translocate to the cell membrane and once again this movement coincided with an increase in mTORC1-dependent S6K1 phosphorylation. Furthermore, this movement was displayed to be a result of a v-ATPase-dependent mechanism as the use of siRNA targeting V0 subunit C of ATP-ase resulted in a reduction in mTORC1 activity and peripheral lysosome content. These data, taken together, suggest that the cellular localization of mTOR-lysosome complexes, rather than the trafficking of mTOR to the lysosome, could be the fundamental regulator of mTORC1 activation. We believe these differing findings are a result of the divergent nutrient-deprivation models used (complete vs milder and more “physiologically relevant”). Therefore, this potential mechanism of mTORC1 activation is more likely to relate to the physiological processes occurring in human skeletal muscle, and consequently deserved further investigation.

### mTOR-Lysosomal Trafficking After Resistance Exercise

**Figure 2:**
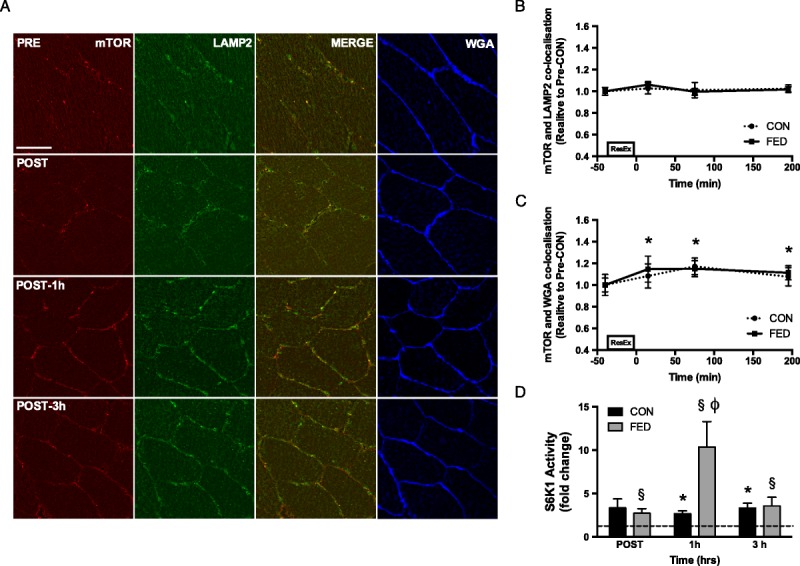
The effect of resistance exercise, with and without protein-carbohydrate feeding, on the mechanistic target of rapamycin (mTOR), the localization with the lysosome (lysosomal-associated membrane protein 2 (LAMP2)), and the cell membrane (WGA) in human skeletal muscle. Representative images are provided (A) with mTOR displayed in red, LAMP2 in green, and WGA in blue. mTOR localization with LAMP2 did not change at any time point in either condition (B). However, mTOR localization with WGA was elevated in both conditions postexercise and remained elevated for 3 h postexercise (C). These changes in mTOR cellular location coincided with elevations in S6K1 kinase activity (D), a common readout of mTORC1 activity. Scale bars are equal to 50 μm. *^§^Significantly different from resting values (*P* < 0.05). ϕSignificant difference between conditions at this time point (*P* < 0.05). All data presented are mean ± SE. [Adapted from (37). CC BY 4.0 (https://creativecommons.org/licenses/by/4.0/).]

Based on the observations from Korolchuk *et al.* (33), our lab investigated the physiological relevance of mTORC1 localization in human skeletal muscle after resistance exercise in the presence (FED) or absence (CON) of protein-carbohydrate feeding (37). To do this, we used immunofluorescence microscopy approaches to identify mTOR-positive structures and lysosomes (LAMP2-positive) and analysed colocalization through the correlation of fluorescence signals. Similar to the findings of Korolchuk *et al.* (33), and contrary to complete starvation protocols (21,30,32), our results display no changes in colocalization of mTOR and the lysosome between the initial postabsorptive period and any postexercise/feeding time point (Figs. 2A, B), reinforcing the notion that during physiological states of nutrient deprivation, mTOR localization at the lysosome is unaffected. We also observed a change in mTOR and LAMP2 colocalization with a marker of the muscle plasma membrane (wheat germ agglutinin (WGA)) after resistance exercise with or without feeding. Specifically, a ~20% increase in both mTOR and LAMP2 colocalization with WGA was noted immediately postexercise (both with and without feeding), and this elevation remained for a further 3 h (Figs. 2A, C). This translocation of mTOR/LAMP2-positive structures was accompanied by a significant increase in S6K1 activity in both subject cohorts (37), with a greater increase apparent in subjects consuming a protein-carbohydrate beverage postexercise (Fig. 2D), a finding consistent with previous data in the field revealing a synergistic effect of exercise and feeding (7). An increase in S6K1 activity is suggestive of a greater phosphorylation status in response to mTOR activation, and results from these kinase assays are proposed to be comparable with immunoblotting techniques targeted toward mTOR activity (S6K1^Thr389^) (38).

In an attempt to further elucidate the role of mTOR-lysosomal trafficking, we next used a within-subject unilateral exercise model to remove any effects of interindividual variability on the findings reported (37). This protocol allowed the comparison between feeding alone and feeding after resistance exercise within an individual simultaneously. Here, we again reported no alteration in mTOR-lysosome colocalization from baseline in either condition; however, a greater colocalization was noted in the FED condition 3 h after exercise/feeding (39). We propose that this may be a result of an increase in lysosomal biogenesis after resistance exercise (40); however, this notion requires further research. mTOR-WGA colocalization increased 1 h postexercise/feeding in both conditions, returning to baseline at 3 h in the FED condition while continuing to rise in the FED + EX condition. Moreover, a significant condition effect was the observed in mTOR-WGA colocalization suggesting that, across the entire timecourse, mTOR localization to the cell periphery was greater in the FED + EX condition (39), thus implying a synergistic effect of resistance exercise and feeding. A trend toward greater colocalization in the FED + EX condition also was observed at the 3-h time point further reinforcing this synergism, and LAMP2-WGA colocalization mirrored this response. Again, these alterations in mTOR-LAMP2 translocation to the cell periphery were accompanied by changes in S6K1 activity, suggesting that the two processes are somewhat related. It is however important to state here that mTOR-LAMP2 translocation, in our work, is not associated directly with S6K1 kinase activity. No difference in mTOR-WGA colocalization, between the two conditions, was observed at 1 h postexercise/feeding, whereas a large difference in S6K1 kinase activity was noted. We believe that this is because mTOR-LAMP2 translocation is not the only factor implicated in mTORC1 activation in human skeletal muscle. Other factors, such as amino acid availability and mechanical transduction, which are likely to be greater in FED + EX conditions, also would affect mTORC1 activity no matter where mTOR-lysosome complexes reside. It is however possible that at later time points (*i.e.*, 3 h post-exercise/feeding), when these additional factors are less prominent, mTOR-lysosome translocation may play a greater role in mTORC1 activation as we have suggested previously (39). Nevertheless, we believe this mTOR-lysosome translocation is a key event in the process of mTORC1 activation, supporting work previously reported *in vitro* (33).

One main limitation with this initial research was that the antibody used targeted the mTOR kinase protein that is present in both mTOR complexes. This resulted in an inability to directly distinguish between the two complexes in colocalization analysis, meaning conclusions relating these measures to mTORC1 activity may not be entirely valid. To combat this, antibodies targeting specific proteins in each mTOR complex (RAPTOR — complex 1, RICTOR — complex 2) were validated and used (39). Colocalization analysis of these proteins revealed that it is indeed mTORC1 that is the complex seemingly translocating in human skeletal muscle. This was concluded as RICTOR positive structures were visualized close to the cell periphery at baseline, and this remained unchanged throughout trials in either condition (39). Moreover, RAPTOR-WGA colocalization increased slightly at 1 h postexercise/feeding in both conditions, returned to baseline in the FED + EX condition at 3 h yet dropped significantly below baseline values in the FED condition (39). The difference between conditions at this 3-h time point also was noted as significant, suggesting that RAPTOR-WGA colocalization was greater in the FED + EX condition similar to the findings noted with mTOR-WGA colocalization. RAPTOR-mTOR colocalization remained unchanged throughout suggesting that these proteins moved in unison, as a complex (mTORC1), toward the cell periphery. These data thus suggest that mTORC1 is the most likely candidate of mTOR translocation in skeletal muscle and validates conclusions made relating mTOR-lysosome translocation to mTORC1 activity. Of note, only one other study has investigated these processes in human skeletal muscle (41). Here, mTOR-LAMP2 colocalization was noted to increase at 3 h after a resistance exercise bout, only in type II fibers. This contrast to our reported findings is most likely due to the analysis method used. In this study, authors disregarded peripheral regions of muscle fibers during analysis despite stronger immunofluorescent staining apparent in these areas compared with intracellular regions. Therefore, it is possible that the intracellular colocalization of mTOR and LAMP2 was observed to increase because only a small proportion of these proteins were actually included in the analysis. Furthermore, as any analysis regarding the translocation of mTOR toward the sarcolemmal membrane was not conducted, we are unable to conclude whether this study’s findings are congruent with our hypothesis. Nevertheless, the purported fiber-type difference in mTOR colocalization is an area that merits future investigations, especially any possible variation in movement toward the cell periphery between differing fibers.

An important detail to discuss here is our use of WGA as a membrane marker. WGA recognizes many glycosylated proteins that are found on the sarcolemmal membrane of skeletal muscle (42) and is noted as a valid sarcolemmal membrane marker (43). Although not the most specific and sensitive marker of this membrane, we believe that the colocalization of this marker and mTOR/LAMP2 is a valid measure, but we are not suggesting direct association of these constructs. This readout of colocalization is used predominantly as an inference of closer association of the proteins investigated, allowing us to display an increased translocation of mTOR-LAMP2 constructs toward the sarcolemmal membrane.

Overall, our recent work proposes that mTORC1/lysosomal translocation toward the cell periphery is a principal event regulating mTORC1 activation after both resistance exercise and nutrient availability in human skeletal muscle.

### Why Do mTOR/Lysosomal Complexes Translocate in Skeletal Muscle?

As mTORC1-lysosome trafficking toward the cell periphery seems to be important for mTORC1 activation in response to several anabolic stimuli, an important question then becomes as to why this process might occur? Our lab has begun to investigate this through the use of immunofluorescence microscopy to identify mTOR protein-protein interactions. We identified mTOR to translocate close to skeletal muscle microvasculature (identified by ulex europaeus agglutinin 1 staining) after resistance exercise (37), both with and without protein-carbohydrate feeding. As such, mTOR appears to move closer to blood vessels after resistance exercise and protein-carbohydrate ingestion, and the influx of the substrates needed for MPS (amino acids) originating from this area (44) may provide a partial explanation as to why the translocation of mTORC1 controls its activity.

The tuberous sclerosis complex 2 (TSC2)-Rheb axis of mTORC1 activation has been studied previously *in vivo* by immunofluorescence microscopy techniques, with eccentric contractions eliciting the translocation of TSC2 away from Rheb at the lysosome membrane in rodent skeletal muscle (27). TSC2 displays GAP activity toward Rheb (45) and, when associated, maintains Rheb in a GDP-loaded state that cannot bind to the catalytic domain of mTOR and influence its activity (28). Our lab studied the mechanism of mTORC1 activation in human skeletal muscle and, intriguingly, we were able to visualize both Rheb and TSC2 close to the sarcolemmal membrane (37). Furthermore, in response to anabolic stimuli (resistance exercise and protein feeding), we report a reduction in Rheb-TSC2 colocalization along with a reciprocal increase in mTOR-Rheb colocalization. These alterations in the cellular location of such proteins indicate a mechanism by which mTORC1 activity could be modulated directly at the cell periphery.

Given that mechanisms of mTORC1 activation appear to converge close to the cell periphery, it is plausible to hypothesize that downstream substrates of mTORC1, or related pathways, also may be located in such areas. As mTORC1 controls cellular translational capacity, we investigated whether mTOR increased interaction with eukaryotic translation initiation factor 3 subunit F (eIF3F), a translational initiation factor that is a component of the ribosome preinitiation complex believed to be essential for the stimulation of protein translation (46). Positive eIF3F puncta were identified close to the cell periphery, and mTOR’s colocalization with this protein was elevated immediately after resistance exercise (37). Interestingly, the interaction between mTOR and eIF3F was greater if resistance exercise was followed by a protein-carbohydrate beverage, compared with the exercise bout in isolation. The overall process of MPS also has been displayed to occur in regions close to the cell periphery, through the use of the antibiotic puromycin (47). Here, positive puromycin staining, indicative of increased protein synthesis, was apparent close to cell borders (48). Overall, these observations provide further clarification as to why mTORC1 translocates after these stimuli yet also elucidates a possible mechanism as to why skeletal muscle loading alongside nutrient provision is able to enhance mTORC1 activity/MPS to a greater extent than either stimulus alone (7).

There also are several other potential candidates for mTOR interaction close to the sarcolemmal membrane, which require further investigation. Protein kinase B, a kinase which phosphorylates TSC2 in response to growth factors, removing its inhibition of Rheb and therefore activating mTORC1, has been visualized at the periphery of HeLa cells (33). Furthermore, the mechanotransducer focal adhesion kinase, implicated in the conversion of mechanical stimuli to mTORC1 activation, is expressed at the periphery of muscle fibers, close to blood vessels (49), an area where mTORC1 is noted after anabolic stimuli (37). Amino acid transporters, as catalysts of the transport of systemic amino acids into muscle, are believed to reside close to blood vessels, allowing the efficient transport of these MPS substrates. Our group recently has confirmed this through the visualization of the primary leucine transporter L-type amino acid transporter 1 close to the skeletal muscle vasculature (50). Finally, there also is an abundance of ribosomal RNA close to the sarcolemma (51) and a subsarcolemmal pool of ribosomes has been identified (52), showing that the site of translation is most likely to be in the region which mTORC1 is located. All of these possible targets of mTORC1 translocation require further investigation to extend the myriad of protein-protein interactions already identified by our group (37) and fully understand the influence of mTORC1-lysosome translocation in human skeletal muscle.

### Future Directions

We propose the identification of mTORC1-lysosome translocation as a mechanism of mTORC1 activation in human skeletal muscle provides novel insight into the molecular regulation of MPS. Currently, to our knowledge, there are only three studies investigating mTOR/lysosomal translocation in human skeletal muscle, and of these, only two measure this complex’s translocation toward the sarcolemmal membrane. Therefore, more research is needed to characterize this process and understand how varying feeding/exercise protocols may affect it. Of these studies, all have been performed in young, healthy men, so the relevance of these findings outside this cohort demographic has yet to be determined. Furthermore, the concurrent use of stable isotope methodology in future studies of these mechanisms is required to confirm the role of this cellular process in the control of MPS. Further research also should be aimed at understanding the mechanisms that drive this mTOR translocation. Currently, it is unclear whether this cellular process contributes to, or is a result of, mTOR activation. Previous work inhibiting lysosomal trafficking (33) reported a complete removal of mTOR activation in response to amino acids, suggesting that this process contributes to the activation of the kinase. However, as we have identified several downstream targets in areas close to where mTOR is translocating toward (37), it is plausible that this intracellular movement may be a result of mTOR activation itself. It also will be of interest to examine the localization of mTORC1/lysosomal complexes in populations that exhibit skeletal muscle anabolic resistance, that is, elderly individuals (53), to determine whether the inhibit MPS observed in these scenarios is due to impaired mTORC1 trafficking after an anabolic stimulus. If this phenomenon is observed, drugs and interventions that influence mTORC1 translocation could be developed as a means to counteract skeletal muscle atrophy.

## CONCLUSIONS

**Figure 3:**
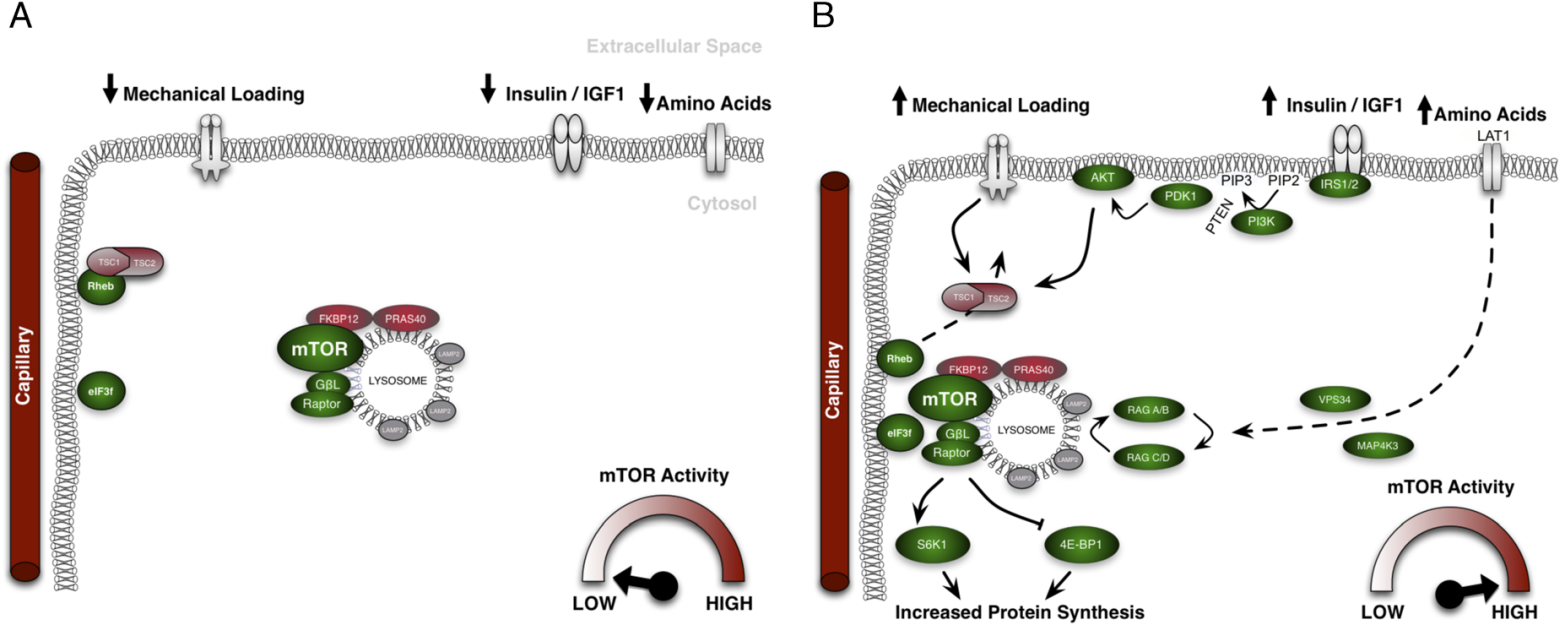
Our proposed model of the mechanistic target of rapamycin complex 1 (mTORC1) activation in human skeletal muscle. In the postprandial, resting state, mTORC1 and the lysosome are associated yet reside in the sarcoplasm of muscle cells. Ras homolog enriched in brain (Rheb), situated close to the cell membrane, is associated with tuberous sclerosis complex 2 (TSC2) and therefore guanosine-diphosphate (GDP)-loaded and inactive. This results in low mTORC1 activity and low levels of muscle protein synthesis (A). Upon the introduction of anabolic stimuli, that is, amino acid ingestion or mechanical stimuli, translocation of mTORC1 (and the lysosome) toward the cell membrane is initiated. Simultaneously, TSC2 becomes phosphorylated and is removed from Rheb, allowing it to become guanosine-triphosphate (GTP)-loaded and active. mTORC1-lysosome complexes associate with Rheb, activating mTORC1 and increasing its activity. Active mTORC1 then resides in areas close to blood vessels, amino acid transporters, and translation initiation factors (eIF3F), which allows a more efficient stimulation of protein synthesis that is elevated subsequently (B).

The importance of mTORC1 activity in stimulating MPS, and therefore skeletal muscle hypertrophy, is accepted and characterized widely. The current, most widely accepted, model of mTORC1 activation suggests that the recruitment of mTORC1 to the lysosome is essential to activate this kinase and stimulate protein translation. Alternatively, we propose that in addition to altered mTOR/lysosomal interaction, mTORC1-lysosome complex trafficking toward the sarcolemmal membrane may be a fundamental process involved in mTORC1 activation in human skeletal muscle (Fig. 3). We propose that this intracellular translocation occurs to position mTORC1 close to the sarcolemmal membrane where an abundance of upstream activators and downstream substrates of mTORC1 seems to reside, that is, Rheb, amino acid transporters, and translation initiation factors (Fig. 3). These findings are important to the field of skeletal muscle physiology as they identify a novel process by which MPS may be coordinated after resistance exercise and amino acid provision.
